# Book Reviews

**Published:** 1913-09

**Authors:** 


### BOOK REVIEWS

Treatment of Fistula-in-ano. By J. A. McMillan, M. D., of Detroit, Mich.

There are three essentials for the operation for this condition:

1st. An incision that will open up every ramification of the fistulous tract.

2nd. The excision of the fibrous tissue which forms its walls.

---

A Further Consideration of Sir Charles Ball's Operation for Intestinal Hemorrhoids. By Alfred J. Zobel, M. D., of San Francisco, Cal.

After a trial of this operation the author of the paper sums up his conclusions as to its value, as follows: That, as a modification of the old ligature operation, it is better than the latter, and at the same time is far superior to the clamp and cautery operation, in that it takes care of and avoids the recurrence of that revoluted anal skin ring which generally becomes markedly edematous immediately after these operations, leaving behind skin tags after the swelling subsides.

In every instance in which the essentials of Ball's technique have been followed out careful the author's results have been exceedingly satisfactory.

The operation is recommended.

